# Behavioral effects of wolf presence on moose habitat selection: testing the landscape of fear hypothesis in an anthropogenic landscape

**DOI:** 10.1007/s00442-021-04984-x

**Published:** 2021-08-22

**Authors:** Håkan Sand, Mark Jamieson, Henrik Andrén, Camilla Wikenros, Joris Cromsigt, Johan Månsson

**Affiliations:** 1 Grimsö Wildlife Research Station, Department of Ecology, Swedish University of Agricultural Sciences, 739 93 Riddarhyttan, Sweden; 2 Department of Wildlife, Fish, and Environmental Studies, Swedish University of Agricultural Sciences, 901 83 Skogsmarksgränd, Umeå, Sweden

**Keywords:** Habitat heterogeneity, Landscape of risk, Predation, Vegetation cover, Open habitat

## Abstract

Landscape of fear refers to the spatial variation in prey perception of predation risk, that under certain conditions, may lead to changes in their behavior. Behavioral responses of prey in relation to large carnivore predation risk have mainly been conducted in areas with low anthropogenic impact. We used long-term data on the distribution of moose in different habitat types in a system characterized by intensive management of all three trophic levels (silviculture, harvest of wolves and moose) to study effects on moose habitat selection resulting from the return of an apex predator, the wolf. We assumed that coursing predators such as wolves will cause an increased risk for moose in some habitat types and tested the hypotheses that moose will avoid open or young forest habitats following wolf establishment. After wolf recolonization, moose reduced their use of one type of open habitat (bog) but there was neither change in the use of the other open habitat type (clear-cut), nor in their use of young forest. Wolf establishment did not influence the use of habitat close to dense habitat when being in open habitats. Thus, the effect of wolves varied among habitat types and there was no unidirectional support for a behavioral effect of wolves’ establishment on moose habitat use. Human-driven habitat heterogeneity, concentration of moose forage to certain habitat types, and the effects of a multiple predator guild on moose may all contribute to the results found. We conclude that the landscape of fear is likely to have weak ecological effects on moose in this system.

**Supplementary Information:**

The online version contains supplementary material available at 10.1007/s00442-021-04984-x.

## Introduction

Landscape of fear refers to the spatial variation in prey perception of predation risk (Laundré et al. 2001; Gaynor et al. 2019). The risk in turn may drive prey towards less risky habitats (Lima and Dill 1990; Creel et al. 2005; Thaker et al. 2011), through movement and habitat selection, or through modulation of their activity towards less risky times of day (Fortin et al. 2005; Fischoff et al. 2007; Creel et al. 2008; Valeix et al. 2009b). These effects may in turn have consequences for the demography of populations, and therefore, also have implications for conservation and management.

In large parts of the world, human persecution has resulted in extirpation of large terrestrial carnivores during the last centuries. In some of these areas, prey species have lost anti-predator behavior, as lack of predators result in selection against such costly behavior (Blumstein and Daniel 2005; Sih et al. 2010). When predators return, prey can swiftly re-gain their former anti-predatory behavior (Hunter and Skinner 1998; Laundré et al. 2001; Berger et al. 2001; Creel and Christianson 2008) but prey may also retain anti-predator behavior despite long periods of predator absence (Chamaillé-Jammes et al. 2013). However, there are several cases where predator recolonization has no or weak effects on the response behavior of prey (Kauffman et al. 2010; Middleton et al. 2013; Samelius et al. 2013; Nicholson et al. 2014). One explanation to such contrasting patterns may be that factors affecting the behavior and distribution of prey differ between studies; e.g., (1) the physical landscape (e.g., vegetation and topography); (2) spatial variation in risk (e.g., cover); and (3) perception of risk among prey (e.g., scent, memory) (Gaynor et al 2019). These factors may in turn vary with spatial scale and intensity of land use that may lead to significant changes in for example habitat fragmentation and landscape structures (Estreguil et al. 2013). However, protected landscapes with no or low degree of human impact may vary in terms of habitat composition and landscape structure resulting in contrasting patterns of prey response to risk of predation (Schmidt and Kuijper 2015). For example, Yellowstone National Park (YNP) in North America is characterized by a higher relative degree of open habitat, e.g., meadows mixed with large forest patches (Newman and Watson 2011) as compared to Białowieża national park in Poland which consists of large continuous homogenous broad leaf forest with few and relatively small open areas (Schmidt and Kuijper 2015).

Until recently, the majority of studies on behavioral responses of prey in relation to large carnivore predation risk have been conducted in protected areas (national parks) (Kuijper et al. 2016; Say-Sallaz et al. 2019). However, large carnivores are now also returning to areas with high human impacts (Chapron 2014; Kuijper et al. 2016). Thus, to achieve a more general understanding, there is a need to extend studies on behavioral responses of prey to environments characterized by different degrees of anthropogenic impact.

Coursing predators such as wolves (*Canis lupus*), which rely on extensive movements for encountering prey, are expected to cause an increased risk for prey in open habitats. This is contrary to stalking predators that generally benefit from concealing vegetation in their attempt to stalk prey (Thacker et al. 2011). Visibility is, therefore, likely to be an important factor to perceived predation risk by prey in areas with a relatively high degree of open habitat. Open areas have, therefore, sometimes been considered to be riskier and avoided by ungulates in the presence of large carnivores (Hamilton et al. 1980; Kunkel and Pletcher 2000; Kauffman et al. 2007; Hebblewhite and Merrill 2009) but results are divergent among studies. For example, in YNP Creel et al. (2005a) showed that elk (*Cervus canadensis*) increased their use of forest areas in the presence of wolves, whereas Fortin et al. (2005) showed that elk increased selection of both open and forested habitats but that selection for conifer forest increased in high wolf-use areas, and Mao et al. (2005) found that elk actually selected for more open habitat in winter following the reintroduction of wolves. More recent studies have shown prey to select for open areas when exposed to large carnivores (Tambling et al. 2012; Kohl et al. 2019; Smith et al. 2019). Re-analyses of data from some of the previous studies (e.g., Fortin et al. 2005) have shown that elk regularly used open areas in high wolf-use areas during daily boats of low wolf activity (Kohl et al. 2018, 2019) and that most elk used open areas even during times of the day when wolves were active (Cusak et al. 2019).

In Europe, roe deer (*Capreolus capreolus*) exposed to Iberian wolf (*Canis lupus signatus*) predation showed a stronger anti-predator response (larger group size) in open relative to dense habitats (Barja and Rosellini 2008). From these studies, it is unclear what type of change in prey behavior, if any, would be expected following the return of a coursing predator, such as the wolf in terms of habitat use.

After more than 150 years of absence, wolves returned to central Scandinavia during the 1980s (Wabakken et al. 2001) and the population was estimated to include 450 individuals during the winter of 2019–2020 (Wabakken et al. 2020). Scandinavia is characterized by an intensive silviculture that results in a landscape mosaic of forest stands in different successional stages, bogs, and rocky outcrops (Estreguil et al. 2013). In this landscape, moose browsing during winter is concentrated to early successional stages (young forest), which provide the highest amounts of browse (Månsson 2009). Moose is the primary prey species of wolves (Sand et al. 2005; 2008). The risk of a moose being killed by wolves differs between habitats and is 10–20 times higher in early successional stages such as clear-cuts (open) and young forest (semi-open to dense) as compared to other habitat types (Gervasi et al. 2013).

So far, little evidence exists for the presence of risk effects on prey behavior in relation to habitat types in Scandinavia, but see Sahlén et al. (2016) for an alternative view. Roe deer do not seem to avoid habitats in which the risk of predation by lynx (*Lynx lynx*) was greatest despite lynx predation causing 65% of known mortalities after lynx re-colonized the area (Samelius et al. 2013). Furthermore, little or no support has been found for the presence of risk effects in a number of previous studies on moose exposed to re-colonizing wolves in Scandinavia in terms of habitat use (Nicholson et al. 2014; van Beek Calkoen et al. 2018) or other behavioral effects (Sand et al. 2006; Wikenros et al. 2016; Månsson et al. 2017). However, one explanation may be that previous studies on wolf risk effects on moose in Scandinavia have not been conducted at an appropriate spatial or temporal level (Kuijper et al. 2013). Such analyses could include a temporal comparison of habitat use within a moose population before and after the return of wolves to detect more subtle changes in behavior (Eriksen et al. 2011; Nicholson et al. 2014).

In this study, we use data from 20 years covering a period both before and after wolf establishment, on the distribution of moose during winter in different habitat types to study a potential effect on moose behavior (habitat selection) resulting from the return of an apex predator, the wolf. Under the assumption that wolf predation risk is an important driver of moose habitat selection we test three alternative but not mutually exclusive predictions: (i) if open areas per se are perceived by moose as posing increased predation risk, they should mainly reduce their time in bogs and clear-cuts in response to the local wolf establishment, and (ii) when moose are in open habitats they should also increase their proximity to habitats providing more cover from visual detection (young and old forest), following the return of wolves. Alternatively, results from a previous study in Scandinavia (Gervasi et al. 2013) suggest that (iii) moose should reduce their time mainly in habitats such as clear-cuts and young forest as these (but not bogs) were found to be most risky with regard to wolf predation.

## Methods

### Study area

The study was conducted within Grimsö Wildlife Research Area (140 km^2^) located in south-central Sweden (59–60° N, 15–16° E, ESM Figure S1), within the southern boreal zone. Elevation in the area ranges from 100 to 150 m above sea level. The area is situated in the southern boreal zone and exhibits average January temperatures of −4 °C to −6 °C and average July temperatures of 15 °C to 16 °C (Wastenson et al. 1995). Winter onsets usually in December and spring in late March and summer conditions occur from the start of May until September (Wastenson et al. 1995). Average precipitation is 600–700 mm with 180–210 mm falling as snow, with an average yearly snow depth of 20–30 cm (Wastenson et al. 1995).

The land is mainly covered by forests (78%) interspersed with bogs, lakes, rivers, farmland and meadows. The forest consists mainly of Norway spruce (*Picea abies*) and Scots pine (*Pinus sylvestris*) intermixed with the deciduous trees, downy birch *(Betula pubescens*), silver birch (*B. pendula*), rowan (*Sorbus aucuparia*), aspen (*Populus tremula*)*,* and willow (*Salix* spp.) as well as juniper (*Juniperus communis*) (Månsson et al. 2007a). The field layer consists mainly of dwarf shrubs, especially bilberry (*Vaccinium myrtillus*) and lingonberry (*Vaccinium vitis-idaea*) on the forested land, with dwarf birch (*Betula nana*) and heather (*Calluna vulgaris*) in the bogs. Most of the land is owned by the state-owned national forest enterprise “Sveaskog” and intensive silviculture occurs which means that forest stands are exposed to thinning at regular time intervals and finally clear cutting when approaching a forest stand age less of than 100 years and followed by self-regeneration mixed with planting of seedlings of pine and spruce (Swenson and Angelstam 1993). Forest stands range in size from 0.5 hectares (ha) to 64 ha with an average size of 6 ha (Månsson et al. 2007b).

The moose population in the study area, as well as throughout the country of Sweden is, and has for a long time period (> 60 years), been exposed to intense human management through harvest. Each fall, hunters in Sweden remove some 25–30% of the pre-harvest moose population (Rönnegård et al. 2008, Jonzén et al. 2013). Two aerial counts of moose were conducted in the study area in 2002 and 2006 showing a population density equal to 1.2 (SE = 0.71) and 0.8 (SE = 0.83) moose per km^2^, respectively (Rönnegård et al. 2008). Roe deer are also present with a population of 5.0 per km^2^ in 2005 (Rönnegård et al. 2008). There is also a small population of wild boar (*Sus scrofa*) in the area (Nordström et al. 2009). Following the recolonization of wolves in central Scandinavia starting in 1980s, the first wolf territory overlapping the study area was established during the winter of 2003/2004 and the two adult wolves were GPS-collared in the winter of 2005. Another territory established in 2009 bordering the former territory and eventually overtook the area in 2009 and the two adult wolves were GPS-collared in 2010. Subsequent replacements and GPS-collaring of adult territorials have occurred in the area resulting in continues presence of wolf packs after 2004 (Wabakken et al. 2004; Nicholson et al. 2014). The relatively small size of the study area (140 km^2^) in relation to the normal size of wolf territories (1000 km^2^, range 250–1600 km^2^, Mattisson et al. 2013) have resulted in that the moose population has been continuously exposed to wolves from 2004 and onwards. No legal harvest of wolves has been performed in this area during the study period. Lynx occur regularly in the area but is not a predator on moose (Andrén and Liberg 2015). Brown bears are very rarely found in the area (Ordiz et al. 2015).

### Data collection

We used a long-term dataset (1997–2016) on moose pellet group counts and landscape features to relate the distribution of moose during winter to different habitats. Moose pellet groups have earlier proven to be a reliable method for studying habitat selection (Månsson et al. 2011). The data include 7 years before and 13 years after the return of wolves. We included, as explanatory variables, landscape features known to affect food availability, such as information on year-specific variation in habitat composition and the amount of forage (cover of Scots pine and deciduous trees within browsing height), in addition to predation risk (openness and distance to dense habitats).

### Moose distribution, forage cover, and moose harvest

Monitoring of the winter distribution of moose has been conducted using permanent circular plots of 100 m^2^ (5.64 m radius). The plots were systematically distributed across the research area by 1 × 1 km^2^ squares (*n* = 32) with 20 plots per square (Figure S1). As a result of gravel roads and water bodies some squares have a total of less than 20 plots (Månsson et al. 2011). A minimum of 10 single pellets was required for the pellet group to be counted. The plots are cleared of old pellets in autumn (September–October) and surveyed in spring (April–May) with a mean accumulation period of 186 days (Månsson et al. 2011). Counts in spring represent the moose population from the preceding dormant season (i.e., late autumn–early spring). In addition to pellet counting, the amount of available forage was estimated for each plot every fifth year (1996; 2001; 2016) to roughly map the relation between relative moose density and their food supply over time. The forage estimate occurred within the same plots but with a radius of 2.52 m (20 m^2^) and only included trees within moose browsing height 0.3–3 m (Månsson 2009). The total estimated coverage (%) of the plot was recorded for aspen, downy birch, silver birch, rowan, juniper, willows and scots pine (Hörnberg 2001). Summing all species’ forage estimates together for the plot can, therefore, tally up to more than 100% (Månsson 2009). In the analyses, we summed the forage cover of Scots pine (the primary moose forage during winter; Cederlund et al. 1980) and the sum of deciduous trees cover (preferred forage; Månsson et al. 2007b). Cover of spruce was excluded as it can be neglected as an important food resource for moose (Kalén and Bergquist 2004; Cederlund et al. 1980). As forage availability was only recorded every fifth year we used a linear interpolation; on the plot level; to achieve an annual proxy for forage availability. Moose hunting within the study area is completely controlled by the research unit and moose harvest data (number of moose shot per year) has been recorded since 1973 (Rönnegård et al. 2008).

### Habitat data and distance to cover

The habitat data were obtained from the Sveaskog forest database (GIS based). Since logging activity is intense in the study area, we created habitat maps for each year to account for yearly changes due to clear cutting and successional development. For each year of the study, new clear-cuts were updated, and previous years’ clear-cuts were transitioned into the young forest stage. Using the coordinates of the sample plots, we were able to spatially link forest composition to density of pellet group. No comparable data on forest composition were achievable for private land and consequently 45 sample plots on private land were excluded from the analyses. We distinguished between four different habitat types, three age classes of forested land (≤ 5, 6–35, > 35 years) and bog. These forest age classes roughly apply to the forest management practices within the area (≤ 5 years include clear-cut, 6–35 pre-commercial thinning stage, > 35 thinning and mature). Furthermore, the forest age and forestry actions roughly reflect moose forage availability within the stands (Månsson 2007a) but also the degree of openness. Clear-cuts (≤ 5 year) and bogs are mainly open habitats with no or sparse tree canopy while young forest (6–35 year) range from semi-open to dense, and older forest (> 35 year) is dense habitat. Other habitat types, e.g., bedrock ridges and power line corridors were excluded because of sample size restrictions (only 15 plots). As a proxy of distance to cover we used the distance (m) from the plots within open habitats (i.e., the analyses only included a sub-sample of all plots, see below) to the nearest stand of young or older forest habitat. The distances to cover were measured using the tool NEAR in ArcMap 10.5.

### Snow and temperature data

Mean winter temperature (December to March) and snow data (snow cover according to number of days with > 10 cm snow) were retrieved from weather stations (*n* = 4) within 70 km of Grimsö and downloaded from Swedish Meteorological and Hydrological Institute (SMHI; https://opendata-download-metobs.smhi.se/explore/; Sala N 59.90, E 16.66 [temperature], Kloten N 59.87 E 15.25, Ön N 59.40 E 15.19, Grythyttan N 59.71 E 14.53 and Västvalla N 59.42 E 15.61 [snow data, average from the 4 stations]).

### Statistical analysis

To investigate the influence of wolf establishment on moose distribution, we used two different analyses based on the response variables: binomial (i.e., presence [at least one moose pellet group in a sample plot] or absence of moose pellet groups within a sample plot) and counts (i.e., number of moose pellet groups within a sample plot). In both analyses, we used generalized linear mixed models (GLMM); logistic regression for the binomial response variable and Poisson regression for the count response variable. All statistical analysis was performed using R, version 3.5.2 (R Core Team 2018). We used the libraries *lme4* (Bates et al. 2015), *MuMIn* (Barton 2019), *car* (Fox and Weisberg 2019), *arm* (Gelman and Su 2018) and *Hmisc* (Harrell 2019) and *pROC* (Robin et al. 2011). Model selection was mainly based on AIC, but we also used AUC (Area under the ROC curve; Robin et al. 2011) and accuracy (proportion correctly classified false negative and true positive) to evaluate the logistic regressions. We used pseudo-R^2^ in the Poisson regressions (Nakagawa and Schielzeth 2013). The total sample size per year was 551 sample plots from 32 squares and there was on average 17 (range 7–21) sample plots per square. Most of the samples were either 0 (80%) or 1 moose pellet groups in sample plot (12%). Thus, only 8% of the sample plots had 2 or more moose pellet groups. Sample plots were nested within squares. Squares and years were used as random factors ([intercept | squares] + [intercept | year]) to account for unmeasured spatial and year-related effects. However, year was not included as random factor in models including snow cover, winter temperature, and moose harvest as the sample size was not large enough to separate between yearly random from fixed effects. The total cover of forage and distance to dense habitat types (for the subset of open habitat types; bog and clear-cut) were log10(x + 1)-transformed, because we expect an exponential declining effect of these factors (Månsson et al. 2012). The log10(x + 1)-transformed forage cover, distance to dense habitat types, snow cover, winter temperature, and moose harvest values were standardized to a mean = 0 and a standard deviation = 1, to improve the convergence in the maximum likelihood estimates. The results are presented on the original scales, i.e., back-transformed. We also tested for the effect of number of years after wolf establishment on moose habitat selection by including year as a factor for the subset from 2004. To test how different variables influenced the probability of moose occurrence and the number of moose pellet groups in the sample plots, we always included the factor wolf presence and its interaction with the variables in the model, in accordance to the hypothesis that moose should respond to the wolf establishment differently in different habitat types. In the models including snow cover, we have also included the interactions between habitat type and snow cover, as Månsson (2009) found different responses by moose to snow cover in different habitat types when partly the same data set was analyzed.

To estimate the within habitat effects of wolf establishment, we combined the reference coefficient (“Factor Wolf (Bog)”) with the coefficient relative to the reference (“Wolf * habitat type”). We tested if the two datasets (probability and number of moose pellet groups) described the moose population changes in the same way, by comparing the two variables in regression on log-scale and used the yearly mean across all sample plots in a given year. If two variables have the same relative change, i.e., a proportional relationship, then the slope will be 1 when both variables are on log-scale. To describe changes in the environment during the study period, we analyzed both how the sample plots were distributed in different habitat types and how available forage changed over time.

## Results

The yearly probability and the number of moose pellet groups within a sample plot was significantly correlated (*p* < 0.0001) and the relationship was not significantly different from a proportional relationship (*p* = 0.21; ESM Figure S2). Here, we report the results of the probability of having moose pellet groups within a sample plot (as the data was dominated [92%] to be either 0 or 1 moose pellet group), whereas the results from the number of moose pellet groups within a sample plot is reported in the ESM. Moose density indices did not significantly differ between the two periods (before and after wolf establishment) in the area. The probability of having moose pellet groups within a sample plot was 0.20 (0.16–0.24, 95% CI) before and 0.18 (0.15–0.22, 95% CI, *p* = 0.37) after wolf established in the area (ESM, Figure S3). The mean number of moose pellets groups within a sample plot was 0.31 (0.24–0.40, 95% CI) before and 0.33 (0.26–0.42, 95% CI, *p* = 0.60) after wolf established in the area (ESM, Figure S3). There were different time trends in the proportion of the four habitat types during the study period (ESM, Figure S4). The proportion of sample plots in young forest out of the total number of plots sampled increased during the study period (*p* < 0.001), whereas plots in old forest decreased (*p* < 0.001). The proportion of sample plots in bog habitat was constant, and there was no significant trend in the proportion of sample plots in clear-cuts (*p* = 0.51). The estimated cover of moose forage of different types also changed over the study period. The cover of pine forage decreased during the study period (*p* < 0.001), whereas the cover of deciduous forage increased (*p* < 0.001), which resulted in non-significant trend in the total cover of pine and deciduous forage (*p* = 0.26, ESM, Figure S5).

**Fig. 1:**
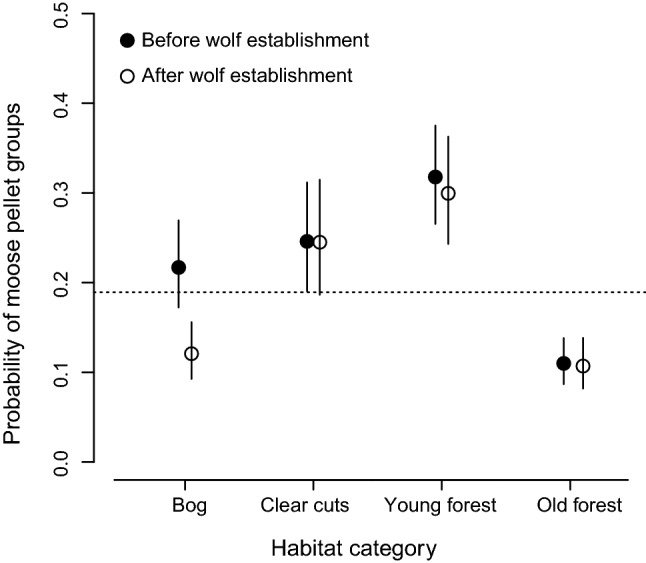
Model prediction and 95% CI of the probability of moose pellet groups in sample plots in the four habitat types before (black dots) and after (open dots) wolf establishment, in Sweden, 1997–2016. The horizontal dotted line indicates the overall probability of having moose pellet groups in sample plots (0.19)

**Fig. 2:**
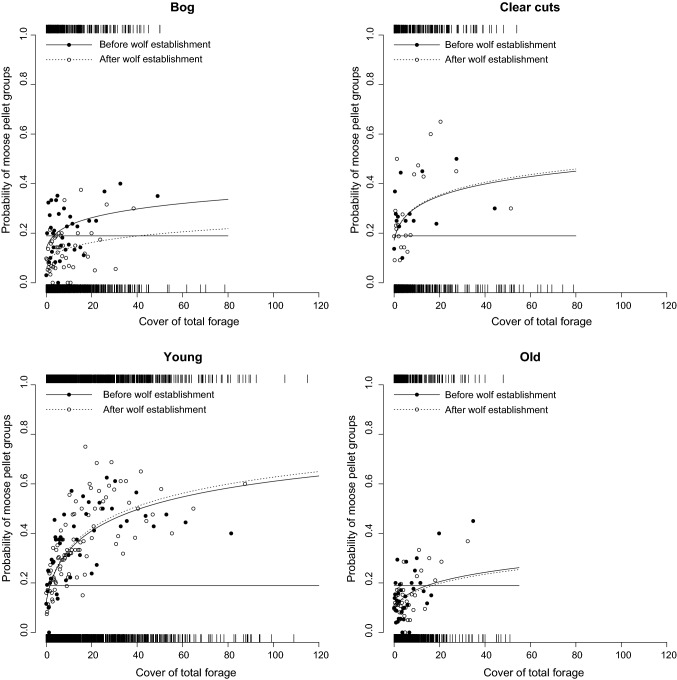
Model prediction probability of moose pellet groups in sample plots in the four habitat types before (black dots and black line) and after (open dots and dotted line) wolf establishment in relation to total forage in Sweden, 1997–2016. The horizontal lines indicate the overall probability of moose pellet groups in sample plots (0.19). Dot values are based on binning the presence/absence (1 and 0) data into groups of 20 samples and estimating the proportion of presence and the mean total forage in the binned group

Both the probability and the number of moose pellet groups within a sample plot differed between the four habitat types, but only the use of bogs changed from before to after wolf established in the area (Fig. 1 and ESM Figure S6). The total cover of moose forage had a strong positive effect on both the probability and the number of moose pellet groups within a sample plot. The most parsimonious model included total forage cover, habitat types, wolf presence, and the interactions between wolf presence and habitat types (W*H), wolf presence and total forage cover (W*F), as well as habitat types and total forage cover (H*F; Table 1). However, the effect size for the interaction wolf presence and total forage cover was only 0.17 (i.e., coefficient/SE, 0.0098/0.059). The interaction between habitat types and total forage cover (H*F) indicates that the slopes between the probability of having moose pellet groups within a sample plot and total forage cover differ between habitat types. The highest slope was in young forest and the lowest slope in bog, but the slopes were not influenced by wolf presence (Table 2, Fig. 2). The models including the effect of snow cover, mean winter temperature, and moose harvest with the interaction with wolf presence had all substantially lower support (Table 1).

**Table 1:** Model selection for explaining the probability of having moose pellet groups within a sample plot

Model	AIC	dAIC	AUC	Accuracy
W + H + F + W*H + W*F ^a^ + H*F	9933.89	0	0.736	0.695
W + H + F + W*H	9947.85	13.96	0.734	0.682
W + H + F + W*H + W*F	9949.65	15.76	0.734	0.682
W + H + F + W*H + S + W*S + H*S ^b^	9953.24	19.35	0.730	0.684
W + H + F + W*H + T + W*T ^b^	9961.23	27.34	0.729	0.680
W + H + F + W*H + M + W*M ^b^	9979.19	45.30	0.728	0.685
W + H + W*H	10,257.65	323.76	0.708	0.647
Null model	10,751.66	817.77	0.645	0.610

The variables included in the models were before and after wolf establishment (W), four habitat types, bog, clear-cut, young forest and old forest (H), total forage (F), snow cover (S), mean winter temperature (T) and moose hunting (M). The variable total forage (F) was log10(x + 1)-transformed and then standardized. The variables snow cover (S), mean winter temperature (T) and moose hunting (M) were standardized. Sample plots were nested under square. Square and year were included as random factors, except in the models including the variables snow cover, winter temperature and moose hunting. The model W + H + F + W*H + W*F + H*F is illustrated in Fig. 2 and the model W + H + W*H is illustrated in Fig. 1. The null model includes only the intercept and the two random factors

^a^The effect size for W*F was 0.164

^b^Year was not included as a random factor together with the variables snow cover, winter temperature and moose hunting as as the sample size was not large enough to separate between yearly random from fixed effects

**Table 2:** Parameter estimates for the best model, i.e., lowest AIC values from Table 1 (W + H + F + W*H + W*F + H*F), for explaining the probability of having moose pellet groups within a sample plot

Variable	Coefficient mean ± SE	*p*	Within habitat effect of wolf	
			Coefficient mean ± SE	*p*
Intercept (bog)^a^	−1.481 ± 0.158	< 0.0001	–	–
Clear-cut	0.341 ± 0.169 ^b^	0.04	–	–
Young forest	0.246 ± 0.134 ^b^	0.002	–	–
Old forest	−0.428 ± 0.138^b^	0.07	–	–
Factor wolf (bog)^a^	−0.621 ± 0.166	0.0002	−0.621 ± 0.166^b^	0.0002
Wolf * clear-cut	0.614 ± 0.209^b^	0.003	−0.008 ± 0.189^c^	0.97
Wolf * young forest	0.578 ± 0.155^b^	0.0002	−0.043 ± 0.161^c^	0.79
Wolf * old forest	0.599 ± 0.168^b^	0.0003	−0.022 ± 0.167^e^	0.89
Total forage (bog)^a^	0.313 ± 0.088	0.0004	–	–
Wolf * total forage	0.0098 ± 0.059	0.87	–	–
Total forage * clear-cut	0.061 ± 0.112^b^	0.55	–	–
Total forage * young forest	0.319 ± 0.091^b^	0.0004	–	–
Total forage * old forest	0.074 ± 0.099^b^	0.46	–	–

Slope total forage	Before	After	*p*^f^
Bog	0.313 ± 0.088	0.323 ± 0.075g	0.91
Clear-cut	0.375 ± 0.101^h^	0.384 ± 0.089^i^	0.92
Young forest	0.632 ± 0.090^j^	0.642 ± 0.089k	0.91
Old forest	0.387 ± 0.094^l^	0.397 ± 0.084m	0.91

Within habitat effect of wolf and the slope of total forage show the changes before to after wolf establishment within the specific habitat. The variable total forage (F) was log10(x + 1)-transformed and then standardized

^a^Bog is the reference

^b^Coefficient in relation to the reference. Within habitat effect of wolf was calculated as:

^c^Clear-cut, mean; − 0.008 = − 0.621 + 0.614 and SE; 0.189 = $\sqrt{({0.166}^{2}+{0.209}^{2})/2}$

^d^Young forest, mean; − 0.0043 = − 0.621 + 0.578 and SE; 0.161 = $\sqrt{({0.166}^{2}+{0.155}^{2})/2}$

^e^Old forest, mean; − 0.022 = − 0.621 + 0.599 and SE; 0.167 = $\sqrt{({0.166}^{2}+{0.168}^{2})/2}$

The slope for “Total forage” before and after wolf establishment was calculated as:

^f^The *p* value indicate the difference in slopes within the habitat type before and after wolf establishment

^g^Bog after, mean; 0.323 = 0.313 + 0.0098 and SE; 0.075 = $\sqrt{({0.088}^{2}+{0.059}^{2})/2}$

^h^Clear-cut before, mean; 0.375 = 0.313 + 0.061 and SE; 0.101 = $\sqrt{({0.088}^{2}+{0.112}^{2})/2}$

^i^Clear-cut after, mean; 0.384 = 0.313 + 0.0098 + 0.061 and SE; 0.089 = $\sqrt{({0.088}^{2}+{0.059}^{2}+{0.112}^{2})/3}$

^j^Young forest before, mean; 0.632 = 0.313 + 0.319 and SE; 0.090 = $\sqrt{({0.088}^{2}+{0.091}^{2})/2}$

^k^Young forest after, mean; 0.642 = 0.313 + 0.0098 + 0.319 and SE; 0.089 = $\sqrt{({0.088}^{2}+{0.059}^{2}+{0.091}^{2})/3}$

^l^Old forest before, mean; 0.387 = 0.313 + 0.074 and SE; 0.094 = $\sqrt{({0.088}^{2}+{0.099}^{2})/2}$

^m^Old forest after, mean; 0.397 = 0.313 + 0.0098 + 0.074 and SE; 0.084 = $\sqrt{({0.088}^{2}+{0.059}^{2}+{0.099}^{2})/3}$

The probability of having moose pellet groups in bogs decreased from before to after wolf establishment (*p* = 0.0002, and Figs. 1, 2, Table 2). In contrast, there were no significant changes for clear-cut (*p* = 0.97), young forest (*p* = 0.79) and old forest (*p* = 0.89). Among the four habitat types young forest was the most selected both before and after wolf establishment with a probability above the overall mean probability of 0.19 (Fig. 1, dotted line). Clear-cut was also selected by moose both before and after wolf establishment. Old forest was not selected and was below the overall mean probability both before and after wolf establishment. Bog was neutral before wolf establishment, as it overlapped the overall mean probability but after wolf establishment, this changed to bogs being avoided. Including the number of years after wolf establishment showed that selection for bog decreased over time (slope = − 0.070 ± 0.023, *p* < 0.003), whereas there were no significant trends in selection in the other habitat types (clear-cut; slope = − 0.011 ± 0.045, *p* = 0.80, young forest; slope − 0.019 ± 0.040, *p* = 0.63, and old forest; slope = − 0.023 ± 0.037, *p* = 0.53).

**Fig. 3:**
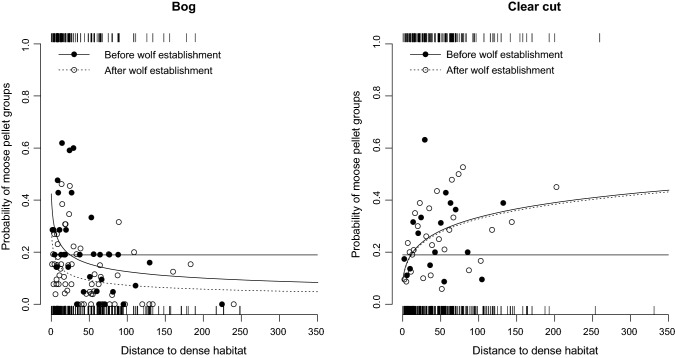
**a** and **b**. Model prediction probability of moose pellet groups in sample plots in the open two habitat types (bog and clear-cut) types before (black dots and black line) and after (open dots and dotted line) wolf establishment in relation to the distance to dense habitat (young and old forest). The predictions are at mean cover of total forage. The horizontal lines indicate the overall probability of in sample plots (0.19). The dots are based on binning the presence/absence (1 and 0) data into groups of 20 samples and estimating the proportion of presence and the mean total forage in the binned group

For moose, use of open habitat types (bog and clear-cut), the most parsimonious model included total forage, habitat types, wolf presence, distance to dense habitat, the interaction between habitat types and wolf presence, and the interaction between distance to dense habitat and habitat types (Tables 3, 4, ESM Table S2), but not the interaction between distance to dense habitat and wolf presence. The probability of having moose pellet groups within a sample plot was influenced by the distance to dense habitat, but the relationship was different between the two habitat types. The probability of having moose pellet groups within a sample plot decreased with distance to dense habitat for bog, whereas it increased for clear-cut (Fig. 3). However, the slopes were not affected by wolf establishment. Finally, snow cover was not significant (*p* = 0.19) in a model also including total forage and distance to dense habitat.

**Table 3:** Model selection for explaining the probability of having moose pellet groups within a sample plot including distance to dense habitat for the subset open habitats (i.e., bog and clear-cut)

Model	AIC	dAIC	AUC	Accuracy
W + H + F + D + W*H + D*H	2597.56	0	0.750	0.687
W + H + F + D + W*D + D*H	2602.11	4.55	0.750	0.694
W + H + F + D + W*H	2642.07	44.51	0.735	0.686
W + H + F + D + W*H + W*D	2642.38	44.82	0.737	0.686
W + H + F + W*H ^a^	2643.00	45.44	0.735	0.678
W + H + F + D + D*H	2646.37	48.81	0.737	0.687
Null model	2719.49	121.93	0.709	0.665

The variables included in the models were before and after wolf establishment (W), two habitat types, bog and clear-cut (H), total forage (F) and distance to dense habitat (D). The variables total forage (F) and distance to dense habitat (D) were log10(x + 1)-transformed and then standardized. Sample plots were nested under square. Square and year were included as random factors. The null model includes only the intercept and the two random factors. The model W + H + F + D + W*H + D*H is illustrated in Fig. 3

^a^The best model without the variable: distance to dense habitat

**Table 4:** Parameter estimates for the best model, i.e., lowest AIC values from Table 3, for explaining the probability of having moose pellet groups within a sample plot, including distance to dense habitat for the subset open habitats (i.e., bog and clear-cut)

Variable	Coefficient mean (± SE)	*p*	Within habitat effect	
			Coefficient mean ± SE	*p*
Intercept (bog)^a^	− 1.521 ± 0.175	< 0.0001	–	–
Clear-cut	0.415 ± 0.186^b^	0.03	–	–
Factor wolf (bog)^a^	− 0.596 ± 0.166	0.0003	− 0.596 ± 0.166^a^	0.0003
Wolf * clear-cut	0.569 ± 0.221^b^	0.01	− 0.027 ± 0.196^c^	0.89
Total forage	0.398 ± 0.055	< 0.0001	–	–
Distance to dense habitat (bog)^a^	− 0.380 ± 0.068	< 0.0001	− 0.380 ± 0.068^a^	< 0.0001
Distance to dense habitat * clear-cut	0.753 ± 0.113^b^	< 0.0001	0.373 ± 0.093^d^	< 0.0001

Within habitat effect of wolf shows the change before to after wolf establishment within the specific habitat. The variables total forage (F) and distance to dense habitat were log10(x + 1)-transformed and then standardized

^a^Bog is the reference

^b^Coefficient in relation to the reference

Within habitat effect calculated as:

^c^Clear-cut, mean; − 0.027 = − 0.596 + 0.569 and SE; 0.196 = $\sqrt{({0.166}^{2}+{0.221}^{2})/2}$

^d^Effect of distance to dense habitat in clear-cut. mean; 0.373 = -0.380 + 0.753 and SE; 0.093 = $\sqrt{({0.068}^{2}+{0.113}^{2})/2}$

In summary, total forage and distance to dense habitat (in the subset of open habitat) explained the probability of having moose pellet groups within a sample plot but the effects of these factors were not affected by wolf presence. Consequently, the interactions between these factors and wolf presence were not included in the best models. Therefore, these relationships have not changed due to wolf establishment. The only wolf-related interaction that was included in one of the best models was the one between habitat types and wolf presence. Thus, the only effect of wolf establishment we could detect was that the probability and number of moose pellet groups decreased within bogs from before to after wolf establishment, whereas there was no significant change in clear-cut, young, and old forest.

## Discussion

In Scandinavia, at least two factors seem to fulfill the critical prerequisites for anti-predator behavioral response of moose to occur. First, there is mix of habitats (e.g., 25% open and 75% dense habitats) and second, predation risk differs among habitats (Gervasi et al. 2013). Our study covered 7 years before wolves established in the study area and 13 years with their presence, a period much longer than covered in most other studies examining behavioral changes of prey in relation to the re-establishment of a large predator. Despite the long-term nature of our study and the high spatial resolution applied for the estimation of moose habitat use, we could not verify any clear and unambiguous effect of wolf establishment in line with any of our three predictions, i.e., in their use of open habitats (i), distances to cover (ii), and to young successional stages (iii). Although moose reduced their use of one open habitat type (bog) after wolf recolonization, there was no change in the use of the other open habitat type (clear-cut) nor in the other two more dense habitats (young and older forest), in our studied landscape. Further, we found different effect of distance to dense habitat on bogs and clear-cut, but neither of them was affected by wolf establishment. Consequently, our first prediction (reduce the time spent in open habitats) was only partly supported by our data and there was no support for our second (decreased distance to habitats providing cover) or third prediction (reduce time spent in riskier habitats; clear-cuts and young forest).

It may appear that the two open habitat types (bogs and clear-cuts) differ in movement resistance since bogs are wet, and therefore, sometimes more energetically costly to move through, which may affect the risk of the two habitat types. However, since our data reflect winter conditions and bogs in general, but not always, are frozen during this season we also tested for a temporal winter effect by including snow depth and winter temperature as an interaction term with wolf presence in our models of habitat use. Neither of these two variables was included in our best model, and therefore, could not explain the decreased use of this type of habitat after wolf establishment. That bogs would be riskier for moose than other habitats is, however, contradicted by a previous study on moose in Scandinavia (Gervasi et al. 2013). In fact, that study showed the other type of open habitat (clear-cuts) in combination with young forest resulted in an increased risk of wolf predation for moose and thus, motivated the formulation of our third prediction, i.e., that these two habitat types should have a lower use by moose after wolf establishment. However, this prediction was also rejected. In addition, results from the current study showed that the wolf variable contributed relatively little to the best models explaining moose habitat use, and that a much larger portion of the variation explained was due to the spatial structuring of sample plots, as indicated by the two random variables included in the analyses. Thus, although we cannot rule out the possibility that wolf establishment has resulted in some type of habitat-related behavioral change in moose, this option seems less likely in the light of the results found by Gervasi et al. (2013).

An alternative explanation for the decreased use of bogs during the latter part of our study period can be the change in the composition of habitats. The proportion of young forest, which was the preferred habitat (Fig. 1), increased during the study period (ESM Figure S4) whereas there was no change in the use of young forest by moose (Table 2) and proxies of moose population size indicated no change before and after wolf establishment (ESM Figure S3). We found a significant decrease in moose use of bogs with increasing availability of young forest (*p* < 0.001), but no significant effect of wolf presence (*p* = 0.64; ESM Figure S7). An increasing proportion of a preferred habitat that is used to the same extent over the study period and a constant population size can result in a decreased use of a less preferred habitat, i.e., a functional response in habitat use (Mysterud and Ims 1998). The decreasing use of bogs by moose is, therefore, likely be an effect of the change in the composition of habitats.

Our results contrast with the pattern found in a number of earlier studies showing an almost instant and clear increase of anti-predator behavior by ungulates when predators return to the environment (Hunter and Skinner 1998; Berger 1999; Berger et al. 2001; Laundré et al. 2001; Creel et al. 2005; Creel and Winnie 2005; Mao et al. 2005). However, our results concur with more recent results from the YNP system that provides no or little support for that wolf establishment will result in less use of open areas or a change in habitat selection (Mao et al. 2005; Proffitt et al. 2009; White et al. 2009; Middleton et al. 2013). Our study also adds to a number of Scandinavian studies that have not found clear evidence for behavioral change in moose or roe deer in relation to the return of their main predator, i.e., wolves or lynx (Gervasi et al. 2013; Samelius et al. 2013; Nicholson et al. 2014; Wikenros et al. 2016; Månsson et al. 2017; van Beeck Calkoen et al. 2018). These contrasting patterns between study areas lead us to ask why these differences exist?

### Differences in landscape heterogeneity may explain contrasting results

Risk effects on prey populations are expected to be more pronounced in landscapes that are heterogeneous because the physical landscape sets the stage for spatial variation in predation risk and associated behavioral trade-offs (Atuo and Connell 2017). For example, YNP provides strong habitat heterogeneity including open land (20–25% meadows) combined with dense forests at higher elevations. Despite this strong habitat heterogeneity, research in the YNP system has been considerable inconsistent regarding if and how wolf predation risk may affect elk habitat selection (Mao et al. 2005; Fortin et al. 2005; Kohl et al. 2018, 2019). However, prey may not be exposed to, or perceive even less, spatially variable predation risk in more homogenous systems similar to mature continuous forests such as Białowieża national park (Schmidt and Kuijper 2015) with small amounts of open habitat (1–3%; Kowalczyk 2010). Given that the proportion of open habitats per se is an important prerequisite for creating a landscape of risk as compared to more dense habitats, the proportion of open habitat in both the Scandinavian system (20–25%; this study) and the YNP system would seem enough to provide a predictable variation in predation risk.

Although our study area may also be considered to be relatively heterogeneous, and with an approximately equal amount of open land to the central parts of YNP, this system is different because intensive silviculture has created a fine grained mosaic of forest stands of different age and successional stages. In our study area, the average size of the four habitat types ranges between 0.036 (bogs) and 0.055 (mature forest) km^2^, respectively. Thus, even though these two ecosystems (YNP and Scandinavia) are similar in the proportion of open habitat they differ markedly in the structure, i.e., the size of habitat patches and how these are distributed in the landscape (Newman and Watson 2011; Månsson et al. 2007b). This may have important implications for the cursorial behavior of wolves and the expression of risk effects on their prey in several ways. Whereas open meadows in YNP will provide elk with the opportunity to visually detect approaching wolves well in advance of an attack, moose in Scandinavia are likely to experience both a lower chance of detecting wolves in the vicinity, but once detected also a shorter distance between predator and prey for escaping an attack. Another interesting aspect is that the site of death of wolf-killed moose may not always be in the same habitat as where the prey first was detected and attacked, but may instead reflect the type of habitat where wolves finally managed to kill the moose attacked. Wikenros et al. (2009) showed that the chasing distance of successful attacks by wolves on moose in Scandinavia was on average 80 m with 90% of all attacks being shorter than 400 m. This means that the site of death would in many cases not be in the same type of habitat as the hunt was initiated by wolves. This could suggest that an adaptive prey behavior would be more linked to a habitat-related escape pattern rather than to a general overall change in habitat selection.

Nevertheless, the results found by Gervasi et al. (2013) suggest that there exists spatial variation in predation risk and that this risk is linked to habitat type. Unfortunately, that study did not make a clear distinction between open and dense habitats since the habitat classes representing the higher risk of mortality included both open (clear-cuts) and forest with semi-open habitat (classified as young forest in this study). Therefore, an adaptive change in moose behavior would be predicted to include a reduction in the use of either or both of the two high-risk habitats (clear-cuts and young forest) combined with an increased use of older forest, a pattern also contradicted by the results in our study.

### No wolf effect on moose selection for edge zones in Scandinavia

Some studies on wolf-prey interactions in YNP also suggest that habitat edges may be riskier than open habitats as wolves have shown to prefer moving along linear features (Bergmann et al. 2006; Kauffmann et al. 2007). As a result, this will lead to an increased risk for prey to be killed along edges or close to linear obstacles in the forest, a pattern also found for wolves in Europe (Kuijper et al. 2013; Bojarska et al. 2017). This pattern could potentially also apply to the Scandinavian forest system in addition to the variable mortality risk observed among habitat types. Although our results showed that distance to young and older forest (in the subset of open habitat) explained some of the variation in the probability of having moose pellet groups within a sample plot, the effect of these factors were not influenced by wolf establishment.

### Variation in resource availability and risk may explain divergent patterns

The possibility for native ungulates to respond to spatial variation in predation risk, after recolonization by a large predator, also depends on the foraging opportunities in habitats of different risk (Lima and Dill 1990; Brown 1999), i.e., how food is distributed among different habitats, and the nutritional condition of prey as animals (McNamara and Houston 1990). In our system, the former may be reflected by clear-cuts and young forest indeed being riskier habitats for moose with regard to wolf predation, but also that the main bulk of important food resources are spatially concentrated to these same habitats. During the winter season, moose rely heavily on Scots pine and birch as the major forage species (Cederlund et al. 1980) which together may constitute ≈70% of all biomass ingested during winter. An estimation of the amount of pine and birch browse available to moose in our study area showed that 75% and 77% was linked to clear-cuts and young forest, respectively. This suggests that although these habitats may impose a higher risk of wolf predation relative to other habitats (Gervasi et al. 2013), moose likely cannot afford alteration in their use of these habitats because of the concentration of food resources during winter to these habitat types (Valeix et al. 2009b; Schmidt and Kuijper 2015). Instead, risk effects may be more likely to occur during the summer season with abundant food resources. However, in a previous study based on GPS-positions of collared moose, where habitat selection was related to predation risk (kernel density estimation based on GPS-tagged wolves), we did not find clear evidence for a behavioral risk effect of wolves neither during summer nor winter (Nicholson et al. 2014). Given the uncertainty of the results, the authors concluded that if wolf risk did influence habitat use by moose, this effect was subtle with the effect slightly more apparent in summer than in winter. Thus, moose are likely to face a trade-off between the increased risk due to the re-appearance of a predator and the spatial concentration of food to certain habitat types during winter when energetic constraints are high. This indicates that human-induced changes in habitat composition through intense silviculture may modify the trade-off between food acquisition and risk avoidance. In the YNP system, this constraint may be illustrated by observed differences in the anti-predator behavior between male and female elk during winter and provides evidence that reduced nutritional condition of the prey are likely to decrease anti-predator responses (Winnie and Creel 2007; Oates et al. 2019).

### The density and type of predators may affect response by prey

An alternative or complementary explanation to the pattern found may stem from a type of *dilution effect* of predation by wolves resulting from a relatively low risk for individual moose to die because of wolf predation as compared to other causes of death. The average moose-to-wolf ratio in our study area has been approximately 200:1 (or 500:1 for adult wolves which are the category responsible for killing moose) based on an average moose density of 1.0 moose/km^2^ during winter and an average wolf territory size of 1000 km^2^ (Rönnegård et al. 2008; Sand et al. 2012; Mattisson et al. 2013). A direct consequence of the relatively low wolf-to-moose ratio is that (i) on average < 10% of the moose in the local population will be killed by wolves annually (Zimmermann 2014; Zimmermann et al. 2019), and (ii) the frequency of encounters between wolves and any individual moose will be low (Eriksen et al. 2008). Wikenros et al. (2016) showed that the average distance between GPS-collared moose and wolves was 11 km in our study area. In addition, 25–30% of the moose population is killed annually due to the combined effect of human harvest and wolf predation meaning that human harvest is responsible for a 2.0–2.5 times higher risk of mortality than wolves (Zimmermann et al. 2019).

### Diel predator activity may be important

Pellet group data have been proven to provide adequate information for habitat use in selection studies (Månsson et al. 2011). However, in our case, it did not provide detailed information about when during the dial cycle animals visited a certain site but rather just a cumulative index over their distribution during the dormant season of the study period. In a recent study, Kohl et al. (2018) showed how the crepuscular activity pattern of wolves in YNP may create a spatio-temporal dynamic landscape of fear for prey (elk) and that elk adjusted use of risky habitats to periods of low wolf activity. Their novel findings show the importance of also considering daily activity patterns and may also partly explain many of the divergent results previously presented on the existence of a landscape of fear, and particularly for wolves and elk in YNP. There is strong empirical support that wolves in Scandinavia exhibit a similar diel activity to the pattern found in YNP, and that the time for killing of moose is concentrated to these activity peaks at dawn and dusk (Sand et al. 2005; Colombo 2013). Therefore, one explanation to the lack of clear support for the landscape of fear hypothesis on moose in our study could be that moose temporally adjusted the use of risky habitats to certain times of low wolf activity. However, this explanation is contradicted by Nicholson et al. (2014) who showed no effect of wolves on moose habitat selection during winter even when accounting for a division of their time into day and night and the fact that the majority (68%) of the wolf-killed moose die during the night in Scandinavia (Colombo 2013).

### Conclusion and perspectives

In a review about the concept of landscape of fear, Gaynor et al. (2019) identified five different conditions when a mismatch between predation risk, risk perception by prey, and their response is likely to exist. These conditions include imperfect information of risk effects by prey, energetic constraints on prey behavior, risk-aversive prey, homogenous predation risk, and the ghost of predation past. Two, or possibly three, of these conditions are likely relevant for Scandinavia and thereby explain why there is no clear difference in habitat selection by moose with and without presence of wolves. First, recent (in an evolutionary perspective) human-induced changes in habitat composition towards increased fragmentation of the landscape is likely to reduce variation in predation risk between habitat types. Although this is not supported by previous studies on habitat-related predation risk (Gervasi et al. 2013), it is not clear what component of moose behavior is expected to respond to the increased risk of wolf predation (habitat use or escape). Second, the concentration of moose winter forage into certain successional stages/habitat types is likely to result in energetic constraints that restrict large-scale alteration of habitat use. Third, the recent recolonization of wolves of central Scandinavia means that prey has been relieved from their predation for a significant period (≈150 years) and that the main cause of mortality instead has been replaced with human harvest. These factors may all be relevant for many areas in the world which have been exposed to high anthropogenic impacts on the landscape and where rates of harvest have replaced predation as the main regulating factor of the prey population for many generations. This and previous studies on the effects of predation (Wikenros et al. 2013, 2015, 2020; Gicquel et al. 2020) and predation risk on wolves and moose in our Scandinavian study area match the general conclusion by Kohl et al. (2018) that the landscape of fear is likely to have weak ecological effects on the ecosystem relative to the direct effects of killing.

## Supplementary Information

Below is the link to the electronic supplementary material.

Supplementary file1 (DOCX 505 KB)

## Data Availability

Data are available at: 10.5061/dryad.ffbg79cvt.

## References

[CR1] Andrén H, Liberg O (2015). Large impact of Eurasian lynx predation on roe deer population dynamics. PLoS ONE.

[CR2] Atuo FA, O’Connell TJ (2017). The landscape of fear as an emergent property of heterogeneity: contrasting patterns of predation risk in grassland ecosystems. Ecol Evol.

[CR3] Barja I, Rosellini S (2008). Does habitat type modify group size in roe deer and red deer under predation risk by Iberian wolves?. Can J Zool.

[CR4] Barton K (2019) MuMIn: multi-model inference. R package version 1.43.6. https://CRAN.R-project.org/package=MuMIn. Accessed 3 May 2021

[CR5] Bates D, Maechler M, Bolker B, Walker S (2015). Fitting linear mixed-effects models using lme4. J Stat Softw.

[CR6] Berger J (1999). Anthropogenic extinction of top carnivores and interspecific animal behaviour: implications of the rapid decoupling of a web involving wolves, bears, moose and ravens. Proc R Soci Lond Ser B Biol Sci.

[CR7] Berger J, Swenson JE, Persson IL (2001). Recolonizing carnivores and naïve prey: conservation lessons from Pleistocene extinctions. Science.

[CR8] Bergman EJ, Garrott RA, Creel S, Borkowski JJ, Jaffe R, Watson EGR (2006). Assessment of prey vulnerability through analysis of wolf movements and kill sites. Ecol Appl.

[CR9] Blumstein DT, Daniel JC (2005). The loss of anti-predator behaviour following isolation on islands. Proc R Soci B Biol Sci.

[CR10] Bojarska K, Kwiatkowska M, Skórka P, Gula R, Theuerkauf J, Okarma H (2017). Anthropogenic environmental traps: Where do wolves kill their prey in a commercial forest?. For Ecol Manage.

[CR11] Brown JS (1999). Vigilance, patch use and habitat selection: foraging under predation risk. Evol Ecol Res.

[CR12] Cederlund G, Ljungqvist H, Markgren G, Stålfelt F (1980). Foods of moose and roe-deer at Grimsö in central Sweden: results of rumen content analyses. Swed Wildl Res (viltrevy).

[CR13] Chamaillé-Jammes S, Malcuit H, Le Saout S, Martin J-L (2014). Innate threat-sensitive foraging: blacktailed deer remain more fearful of wolf than of the less dangerous black bear even after 100 years of wolf absence. Oecologia.

[CR14] Chapron G (2014). Recovery of large carnivores in Europe’s modern human-dominated landscapes. Science.

[CR15] Colombo M (2013) Determinants of winter kill rates of wolves in Scandinavia. Master thesis 2013:16. Swedish University of Agricultural Sciences

[CR16] Creel S, Winnie J, Maxwell B, Hamlin K, Creel M (2005). Elk alter habitat selection as an antipredator response to wolves. Ecology.

[CR17] Creel S, Winnie JA (2005). Responses of elk herd size to fine-scale spatial and temporal variation in the risk of predation by wolves. Anim Behav.

[CR18] Creel S, Winnie JA, Christianson D, Liley S (2008). Time and space in general models of antipredator response: tests with wolves and elk. Anim Behav.

[CR19] Creel S, Christianson D (2008). Relationships between direct predation and risk effects. Trends Ecol Evol.

[CR20] Cusack JJ, Kohl MT, Metz MC, Coulson T, Stahler DR, Smith DW, MacNulty DR (2020). Weak spatiotemporal response of prey to predation risk in a freely interacting system. J Anim Ecol.

[CR21] Eriksen A, Wabakken P, Zimmermann B, Andreassen HP, Arnemo JM, Gundersen H, Milner JM, Liberg O, Linnell J, Pedersen HC, Sand H, Solberg EJ, Storaas T (2008). Encounter frequencies between GPS-collared wolves (*Canis lupus*) and moose (*Alces alces*) in a Scandinavian wolf territory. Ecol Res.

[CR22] Eriksen A, Wabakken P, Zimmermann B, Andreassen H, Arnemo JM, Gundersen H, Liberg O, Linnell J, Milner JM, Pedersen HC, Sand H, Solberg EJ, Storaas T (2011). Activity patterns of predator and prey: a simultaneous study of GPS-collared wolves and moose. Anim Behav.

[CR23] Estreguil C, Caudullo G, de Rigo D, San Miguel J (2013). Forest landscape in Europe: pattern, fragmentation, and connectivity.

[CR24] Fischhoff IR, Sundaresan SR, Cordingley J (2007). Habitat use and movements of plains zebra (*Equus burchelli*) in response to predation in danger from lions. Behav Ecol.

[CR25] Fortin D, Beyer HL, Boyce MS, Smith DW, Duchesne T, Mao JS (2005). Wolves influence elk movements: behaviour shapes a trophic cascade in Yellowstone National Park. Ecology.

[CR26] Fox J, Weisberg S (2019) An {R} Companion to Applied Regression, Third Edition. Thousand Oaks CA: Sage. https://socialsciences.mcmaster.ca/jfox/Books/Companion/. Accessed 3 May 2021

[CR27] Gaynor K, Brown JS, Middleton AD, Power ME, Brashares JS (2019). Landscapes of fear: spatial patterns of risk perception and response. Trends Ecol Evol.

[CR28] Gelman A, Su Y-S (2018) Data analysis using regression and multilevel/hierarchical models. R package version 1.10–1. https://CRAN.R-project.org/package=arm. Accessed 3 May 2021

[CR29] Gervasi V, Sand H, Zimmermann B, Mattisson J, Wabakken P, Linell JD (2013). Landscape structure disentangles predation risk in two sympatric ungulates during wolf re-colonization. Ecol Appl.

[CR30] Gicquel M, Sand H, Månsson J, Wallgren M, Wikenros C (2020). Does recolonization of wolves affect moose browsing damage on young Scots pine?. Forest Ecol Manag.

[CR31] Hamilton GD, Drysdale PD, Euler DL (1980). Moose winter browsing patterns on clear-cuttings in northern Ontario. Can J Zool.

[CR32] Harrell Jr FE (2019) Hmisc: harrell miscellaneous. R package version 4.2–0. https://CRAN.R-project.org/package=Hmisc. Accessed 3 May 2021

[CR33] Hebblewhite M, Merrill EH (2009). Trade-offs between predation risk and forage differ between migrant strategies in a migratory ungulate. Ecology.

[CR34] Hunter LTB, Skinner JD (1998). Vigilance behaviour in African ungulates: the role of predation pressure. Behaviour.

[CR35] Hörnberg S (2001). Changes in population density of moose (*Alces alces*) and damage to forests in Sweden. For Ecol Manage.

[CR36] Kalén C, Bergquist J (2004). Forage availability for moose of young silver birch and Scots pine. For Ecol Manag.

[CR37] Kauffman MJ, Smith VN, Stahler DW, MacNulty DR, Boyce MS (2007). Landscape heterogeneity shapes predation in a newly restored predator–prey system. Ecol Lett.

[CR38] Kauffman MJ, Brodie JF, Jules ES (2010). Are wolves saving Yellowstone’s aspen? A landscape-level test of a behaviorally mediated trophic cascade. Ecology.

[CR39] Kohl MT, Stahler DR, Metz MC, Forester JD, Kauffman MJ, Varley N, White PJ, Smith DW, MacNulty DR (2018). Diel predator activity drives a dynamic landscape of fear. Ecol Monogr.

[CR40] Kohl MT, Ruth TK, Metz MC, Stahler DR, Smith DW, White PJ, Macnulty DR (2019). Do prey select for vacant hunting domains to minimize a multi-predator threat?. Ecol Lett.

[CR41] Kowalczyk R (2010) European bison—the king of the forest or meadows and river valleys? In: Kowalczyk R, Ławreszuk D, Wójcik MJ (eds) European bison conservation in the Białowieża Primeval Forest Threats and prospects of the population development. Mammal Research Institute PAS, Białowieża, pp 123–134 [in Polish]

[CR42] Kuijper DPJ, de Kleine C, Churski M, van Hooft P, Bubnicki J, Jedrzejewska B (2013). Landscape of fear in Europe: wolves affect spatial patterns of ungulate browsing in Białowiez˙a Primeval Forest, Poland. Ecography.

[CR43] Kuijper DP, Sahlén E, Elmhagen B, Chamaillé-Jammes S, Sand H, Lone K, Cromsigt JPGM (2016) Paws without claws? Ecological effects of large carnivores in anthropogenic landscapes. Proc R Soci Lond Ser B Biol Sci 283(1841) 10.1098/rspb.2016.1625 PMC5095381 27798302

[CR44] Kunkel KE, Pletscher DH (2000). Habitat factors affecting vulnerability of moose to predation by wolves in southeastern British Columbia. Can J Zool.

[CR45] Laundré JW, Hernández L, Altendorf KB (2001). Wolves, elk, and bison: reestablishing the Blandscape of fear in Yellowstone National Park, USA. Can J Zool.

[CR46] Lima SL, Dill LM (1990). Behavioral decisions made under the risk of predation: a review and prospectus. Can J Zool.

[CR47] McNamara JM, Houston AI (1990). The value of fat reserves and the tradeoff between starvation and predation. Acta Biotheor.

[CR48] Mao JS, Boyce MS, Smith DW, Singer FJ, Vales DJ, Vore JM, Merrill EH (2005). Habitat selection by elk before and after wolf reintroduction into Yellowstone National Park. J Wildl Manag.

[CR49] Mathisen KM, Milner JM, Skarpe C (2017). Moose–tree interactions: rebrowsing is common across tree species. BMC Ecol.

[CR50] Mattisson J, Sand H, Wabakken P, Gervasi V, Liberg O, Linnell JD, Rauset GR, Pedersen HC (2013). Home range size variation in a recovering wolf population: evaluating the effect of environmental, demographic, and social factors. Oecologia.

[CR51] Middleton AD, Kauffman MJ, McWhirter DE, Jimenez MD, Cook RC, Cook JG, Albeke SE, Sawyer H, White PJ (2013). Linking anti-predator behaviour to prey demography reveals limited risk effects of an actively hunting large carnivore. Ecol Lett.

[CR52] Månsson J, Andren H, Pehrson A, Bergström R (2007). Moose browsing and forage availability: a scale-dependent relationship?. Can J Zool.

[CR53] Månsson J, Kalén C, Kjellander P, Andrén H, Smith H (2007). Quantitative estimates of tree species selectivity by moose (*Alces alces*) in a forest landscape. Scand J for Res.

[CR54] Månsson J (2009). Environmental variation and moose *Alces alces* density as determinants of spatio-temporal heterogeneity in browsing. Ecography.

[CR55] Månsson J, Andrén H, Sand H (2011). Can pellet counts be used to accurately describe habitat selection in ungulates?. Eur J Wildl Res.

[CR56] Månsson J, Bunnefeld N, Andren H (2012). Spatial and temporal predictions of moose winter distribution. Oec.

[CR57] Månsson J, Prima M-C, Nicholson KL, Wikenros C, Sand H (2017). Group or ungroup–moose behavioural response to recolonization of wolves. Front Zool.

[CR58] Nakagawa S, Schielzeth H (2013). A general and simple method for obtaining R^2^ from generalized linear mixed-effects models. Methods Ecol Evol.

[CR59] Newman WB, Watson FGR (2011) The Central Yellowstone Landscape: Terrain, Geology, Climate, Vegetation. Chapter 2. In: Garrott R, White PJ, Watson F (eds) The Ecology of Large Mammals in Central Yellowstone

[CR60] Nicholson KL, Milleret C, Månsson J, Sand H (2014). Testing the risk of predation hypothesis: the influence of recolonizing wolves on habitat use by moose. Oecologia.

[CR61] Nordström J, Kjellander P, Andren H, Mysterud A (2009). Can supplemental feeding of red foxes *Vulpes vulpes* increase roe deer *Capreolus capreolus* recruitment in the boreal forest?. Wildl Biol.

[CR62] Oates BA, Merkle JA, Kauffman MJ, Dewey SR, Jimenez MD, Vartanian JM, Becker SA, Goheen JR (2019). Antipredator response diminishes during periods of resource deficit for a large herbivore. Ecology.

[CR63] Ordiz A, Milleret C, Kindberg J, Månsson J, Wabakken P, Swenson JE, Sand H (2015). Wolves, people, and brown bears influence the expansion of the recolonizing wolf population in Scandinavia. Ecosphere.

[CR64] Proffitt KM, Grigg JL, Hamlin KL, Garrott RA (2009). Contrasting effects of wolves and human hunters on elk behavioral responses to predation risk. J Wildl Manag.

[CR65] R Core Team (2018). R: a language and environment for statistical computing. R foundation for statistical computing, Vienna, Austria. https://www.R-project.org/. Accessed 3 May 2021

[CR66] Rönnegård L, Sand H, Andrén H, Månsson J, Pehrson Å (2008). Evaluation of four methods used to estimate population density of moose (Alces alces). Wildl Biol.

[CR67] Sahlén E, Noell S, DePerno CS, Kindberg J, Spong G, Cromsigt JPGM (2016). Phantoms of the forest: legacy risk effects of a regionally extinct large carnivore. Ecol Evol.

[CR68] Samelius G, Andrén H, Kjellander P, Liberg O (2013). Habitat selection and risk of predation: re-colonization by lynx had limited impact on habitat selection by Roe Deer. PLoS ONE.

[CR69] Sand H, Zimmermann B, Wabakken P, Andrén H, Pedersen HC (2005). Using GPS-technology and GIS-cluster analyses to estimate kill rates in wolf-ungulate ecosystems. Wildl Soc Bull.

[CR70] Sand H, Wikenros C, Wabakken P, Liberg O (2006). Cross continental differences in patterns of predation: will naïve moose in Scandinavia ever learn? Royal society of London. Proc Biol Sci.

[CR71] Sand H, Wabakken P, Zimmermann B, Johansson O, Pedersen HC, Liberg O (2008). Summer kill rates and predation pattern in a wolf–moose system: can we rely on winter estimates?. Oecologia.

[CR72] Sand H, Vucetich JA, Zimmermann B, Wabakken P, Wikenros C, Pedersen HC, Peterson RO, Liberg O (2012). Assessing the influence of prey-predator ratio, prey age structure and packs size on wolf kill rates. Oikos.

[CR73] Say-Sallaz E, Chamaillé-Jammes S, Fritz H, Valeix M (2019). Non-consumptive effects of predation in large terrestrial mammals: mapping our knowledge and revealing the tip of the iceberg. Biol Cons.

[CR74] Schmidt K, Kuijper DPJ (2015). A “death trap” in the landscape of fear. Mamm Res.

[CR75] Sih A, Barbosa P, Castellanos I (2005). Predator–prey space use as an emergent outcome of a behavioral response race. Ecology of predator–prey interactions.

[CR76] Sih A, Bolnick DI, Luttbeg B, Orrock JL, Peacor SD, Pintor LM, Preisser E, Rehage JS, Vonesh JR (2010). Predator-prey naivete, antipredator behavior, and the ecology of predator invasions. Oikos.

[CR77] Shi J, Li D, Xiao W (2010). Influences of sex, group size, and spatial position on vigilance behavior of Przewalski’s gazelles. Acta Theriol.

[CR78] Smith JA, Donadio E, Pauli JN, Sheriff MJ, Bidder OR, Middleton AD (2019). Habitat complexity mediates the predator–prey space race. Ecology.

[CR79] Svensson L, Wabakken P, Maartmann E, Åkesson M, Flagstad Ø, Hedmark E (2020) Inventering av varg vintern 2019–2020. Bestandsovervåking av ulv vinteren 2018–2019. Bestandsstatus for store rovdyr i Skandinavia. Beståndsstatus för stora rovdjur i Skandinavien. Report 1–2019, pp 53 (in Swedish)

[CR80] Swenson JE, Angelstam P (1993). Habitat separation by sympatric forest grouse in Fennoscandia in relation to boreal forest succession. Can J Zool.

[CR81] Tambling CJ, Druce DJ, Hayward MW, Castley JG, Adendorff J, Kerley GI (2012). Spatial and temporal changes in group dynamics and range use enable anti-predator responses in African buffalo. Ecology.

[CR82] Thaker M, Vanak AT, Owen CR, Ogden MB, Niemann SM, Slotow R (2011). Minimizing predation risk in a landscape of multiple predators: effects on the spatial distribution of African ungulates. Ecology.

[CR83] Valeix M, Fritz H, Loveridge A, Davidson Z, Hunt J, Murindagomo F, Macdonald D (2009). Does the risk of encountering lions influence African herbivore behaviour at waterholes?. Behav Ecol Sociobiol.

[CR84] Valeix M, Loveridge AJ, Chamaillé-Jammes S, Davidson Z, Murindagomo F, Fritz H, Macdonald DW (2009). Behavioral adjustments of African herbivores to predation risk by lions: spatiotemporal variations influence habitat use. Ecology.

[CR85] Valeix M, Loveridge A, Davidson Z, Madzikanda H, Fritz H, Macdonald D (2010). How key habitat features influence large terrestrial carnivore movements: waterholes and African lions in a semi-arid savanna of north-western Zimbabwe. Landsc Ecol.

[CR86] van Beeck Calkoen STS, Kuijper DPJ, Sand H, Singh NJ, van Wieren SE, Cromsigt JPGM (2018). Does wolf presence reduce moose browsing intensity in young forest plantations?. Ecography.

[CR87] Wabakken P, Sand H, Liberg O, Bjärvall A (2001). The recovery, distribution, and population dynamics of wolves on the Scandinavian peninsula, 1978–1998. Can J Zool.

[CR88] Wabakken P, Aronson Å, Sand H, Strømseth T, Kojola I (2004) Ulv i Skandinavia: Statusrapport for vinteren 2003–2004. Høgskolen i Hedmark, Report nr. 5-2004. 41 pp (in Norwegian)

[CR89] Wastenson L, Raab B, Vedin H (1995) National Atlas of Sweden: Climate, Lakes and Rivers. Chapters, Air Temperature pg 44 (Raab and Vedin) and Precipitation and Thunderstroms pg 76 (Alexandersson and Andersson), Swedish Meterorological and Hydrological Institute, Almqvist and Wiksell International Stockholm

[CR90] White PJ, Garrott RA, Cherry S, Watson FGR, Gower CN, Becker MS, Meredith E, Garrott RA, White PJ, Watson GR (2009). Changes in elk resource selection and distribution with the reestablishment of wolf predation risk. The ecology of large mammals in central Yellowstone: sixteen years of integrated field studies.

[CR91] Wikenros C, Sand H, Wabakken P, Liberg O, Pedersen H-C (2009). Wolf predation on moose and roe deer: chase distances and outcome of encounters. Acta Theriologia.

[CR92] Wikenros C, Sand H, Ahlqvist P, Liberg O (2013). Biomass flow and scavengers use of carcasses after re-colonization of an apex predator. PLoS ONE.

[CR93] Wikenros C, Sand H, Bergström R, Liberg O, Chapron G (2015). Moose hunters adaptively compensates for predation following wolf return in Sweden. PLoS ONE.

[CR94] Wikenros C, Balogh G, Sand H, Nicholson KL, Månsson J (2016). Mobility of moose–comparing the effects of wolf predation risk, reproductive status and seasonality. Ecol Evol.

[CR95] Wikenros C, Sand H, Månsson J, Maartmann E, Eriksen A, Wabakken P, Zimmermann B (2020). Impact of a recolonizing, cross-border carnivore population on ungulate harvest in Scandinavia. Sci Rep.

[CR96] Winnie J, Creel S (2007). Sex-specific behavioural responses of elk to spatial and temporal variation in the threat of wolf predation. Anim Behav.

[CR97] Zimmermann B (2014). Predatory behaviour of wolves in Scandinavia.

[CR98] Zimmermann B, Nelson L, Wabakken P, Sand H, Liberg O (2014). Behavioral responses of wolves to roads: Scale-dependent ambivalence. Behav Ecol.

[CR99] Zimmermann B, Wikenros C, Sand H, Eriksen A, Wabakken P (2019) Moose in wolf territories: predation and hunter harvest. (In Norwegian with english summary). Report nr. 23–2019, Høgskolen i Innlandet, Elverum, Norway. 50 pp

