# Supplementary material for: Behavioral effects of wolf presence on moose habitat selection: testing the landscape of fear hypothesis in an anthropogenic landscape
Source: Oecologia. 2021 Aug 22;197(1):101–16. doi: 10.1007/s00442-021-04984-x (PMC8445880; doi:10.1007/s00442-021-04984-x)
Supplement: Supplementary file 1 — Supplementary file1 (DOCX 505 KB) [file 442_2021_4984_MOESM1_ESM.docx]

Electronic Supplemental Material

Figure S1


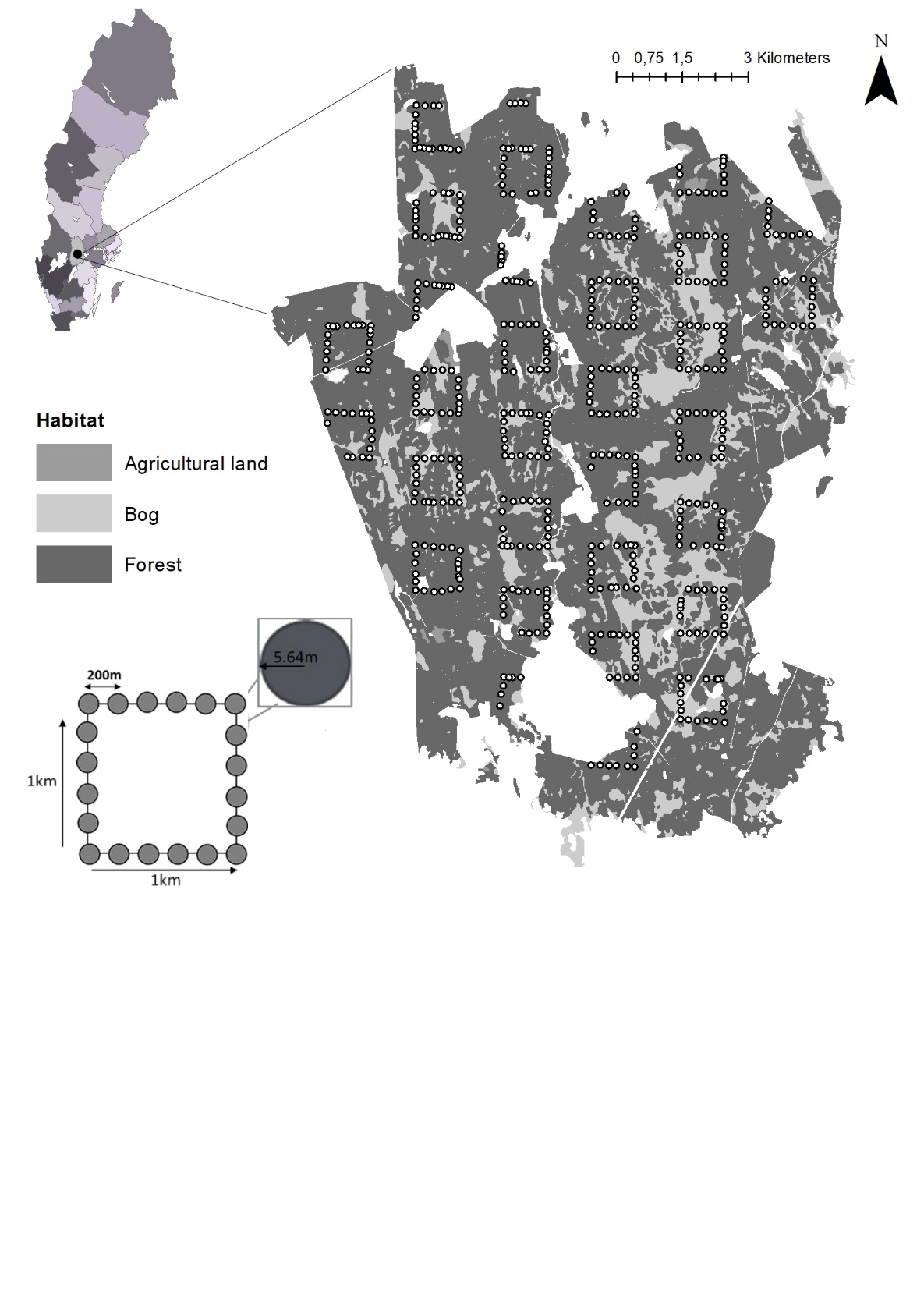


Figure S1. The location of the Grimsö Wildlife Research Area within Sweden, habitat composition (white is private land with missing data) and the distribution of the plots used for data collection of moose pellet groups (radius 5.64 m) and forage availability (radius 2.52 m) 1996/1997 – 2016.

Figure S2

sssss

Figure S2. Relationship between yearly log(probability of plots with moose pellet groups within a sample plot) and log(number of moose pellet groups within a sample plot). The correlation between them was r = 0.836, df = 18, p < 0.0001 and the relationship (Y = 0.94 (± 0.31) + 1.21 (± 0.19) * X) was not significantly different from a proportional relationship (slope = 1 in a log-log relationship, indicated with the red line; t = 1.09, df = 18, p = 0.21). A proportion relationship means the same relative change in both variables, e.g. a 10 % increase in one of the variables corresponds to a 10 % increase in the other variable.

Figure S3

Figure S3. Probability (left) and number (right) of moose pellet groups in sample plots in relation to year in the Grimsö Wildlife Research Area. Wolf establishment indicated by the vertical dotted line. The horizontal lines indicate the overall probability (left) and overall number (right) of moose pellets groups in sample plots before (1997 – 2003) and after (2004 – 2016) wolf establishment.

Figure S4

Figure S4. The proportion of the four habitat types in relation to year during the study period in the Grimsö Wildlife Research Area. Wolf establishment is indicated by the vertical dotted line. The sample size was 551 plots per year.

Figure S5

Figure S5. Box plot of the estimated total forage available to moose in relation to year. Wolf established in the area between 2003 and 2004.

Figure S6

Figure S6. Model prediction and 95% CI of the number of moose pellet groups in sample plots in the four habitat types before (black dots) and after (open dots) wolf establishment, in Sweden, 1997-2016. The horizontal dotted line indicates the overall number of moose pellet groups in sample plots (0.32).

Figure S7

Figure S7. Yearly proportion of plots in bogs with moose pellet groups in relation to the yearly proportion of plots in young forest. Black dots before wolf establishment and open dots after wolf establishment. There was no significant effect of wolf presence (p = 0.64), but a significant negative relationship between yearly proportion of plots in bogs with moose pellet groups and the yearly proportion of plots in young forest (p < 0.001).

Table S1. Model selection for explaining the number of moose pellet groups within a sample plot. The variables included in the models were before and after wolf establishment (W), four habitat types, bog, clear cut, young forest and old forest (H), total forage (F), snow cover (S), mean winter temperature (T) and moose hunting (M). The variable total forage (F) was log10(x+1)-transformed and then standardized. The variables snow cover (S), mean winter temperature (T) and moose hunting (M) were standardized. Sample plots were nested under square. Square and year were included as random factors, except in the models including the variables snow cover, winter temperature and moose hunting. The model W+H+W*H is illustrated in ESM Figure S6. The null model includes only the intercept and the two random factors.

| Model | AIC | dAIC | Pseudo R^2^ fixed factors | Pseudo R^2^ random factors |
| --- | --- | --- | --- | --- |
| W+H+F+W*H+W*F+H*F | 17221.15 | 0 | 0.154 | 0.078 |
| W+H+F+W*H+W*F | 17268.87 | 47.72 | 0.170 | 0.076 |
| W+H+F+W*H | 17272.80 | 51.65 | 0.169 | 0.077 |
| W+H+F+W*H+S+W*S+H*S ^a^ | 17316.83 | 95.68 | 0.177 | 0.059 |
| W+H+F+W*H+T+W*T ^a^ | 17369.39 | 148.24 | 0.176 | 0.059 |
| W+H+F+W*H+M+W*M ^a^ | 17472.80 | 251.65 | 0.170 | 0.059 |
| W+H+W*H | 18186.82 | 965.67 | 0.121 | 0.082 |
| Null model | 19584.31 | 2363.16 | 0 | 0.118 |

^a^ Year was not included as a random factor, as the variables snow cover, winter temperature and moose hunting have the same value within a year.

Table S2. Model selection for explaining the number of moose pellet groups within a sample plot including distance to dense habitat for the subset open habitats (i.e. bog and clear cut). The variables included in the models were before and after wolf establishment (W), two habitat types, bog and clear cut (H), total forage (F) and distance to dense habitat (D). The variables total forage (F) and distance to dense habitat (D) were log10(x+1)-transformed and then standardized. Sample plots were nested under square. Square and year were included as random factors. The null model includes only the intercept and the two random factors.

| Model | AIC | dAIC | Pseudo R^2^ fixed factors | Pseudo R^2^ random factors |
| --- | --- | --- | --- | --- |
| W+H+F+D+W*H+D*H | 4085.48 | 0 | 0.094 | 0.209 |
| W+H+F+D+W*D+D*H | 4092.54 | 7.06 | 0.089 | 0.204 |
| W+H+F+W*H ^a^ | 4130.59 | 45.11 | 0.069 | 0.189 |
| W+H+F+D+D*H | 4131.51 | 46.03 | 0.069 | 0.189 |
| W+H+F+D+W*H | 4136.88 | 51.40 | 0.065 | 0.187 |
| W+H+F+D+W*H+W*D | 4137.08 | 51.60 | 0.064 | 0.190 |
| Null model | 4259.68 | 174.20 | 0 | 0.163 |

^a^ The best model without the variable distance to dense habitat.

Table S3. Descriptive statistic of the variables included in the models.

| Variable | Mean ± SD | Range |
| --- | --- | --- |
| Total forage | 7.05 ± 11.70 | 0 – 115 |
| log10(total forage+1) | 0.578 ± 0.520 | 0 – 2.06 |
| Standardized log10(total forage+1) | 0 ± 1 | -1.11 – 2.86 |
| Snow cover (number of days > 10 cm snow) | 78 ± 35 | 13 – 131 |
| Standardized snow cover | 0 ± 1 | -1.87 – 1.51 |
| Mean winter temperature (°C) December to March | -2.1 ± 1.8 | -6.4 – 0.8 |
| Standardized mean winter temperature | 0 ± 1 | -2.37 – 1.60 |
| Moose hunting bag (number of moose) | 33 ± 14 | 11 – 55 |
| Standardized moose hunting bag | 0 ± 1 | -1.53 – 1.88 |
|  |  |  |
| *Subset open habitats (bog and clear cut)* |  |  |
| Total forage | 7.30 ± 9.80 | 0 – 79 |
| log10(total forage+1) | 0.676 ± 0.465 | 0 – 1.90 |
| Standardized log10(total forage+1) | 0 ± 1 | -1.45 – 2.64 |
| Distance to dense habitat (m) | 161 ± 136 | 0 – 875 |
| log10(distance to dense habitat+1) | 2.02 ± 0.47 | 0 – 2.94 |
| Standardized log10(distance to dense habitat+1) | 0 ± 1 | -4.22 – 1.95 |
